# GCViT: a method for interactive, genome-wide visualization of resequencing and SNP array data

**DOI:** 10.1186/s12864-020-07217-2

**Published:** 2020-11-23

**Authors:** Andrew P. Wilkey, Anne V. Brown, Steven B. Cannon, Ethalinda K. S. Cannon

**Affiliations:** 1 ORISE Fellow, USDA-ARS Corn Insects and Crop Genetics Research Unit, Ames, IA 50011 USA; 2 USDA-ARS Corn Insects and Crop Genetics Research Unit, Ames, IA 50011 USA

**Keywords:** GCViT, CViT, SNP, Resequencing, Genotype, Visualization, UI, Web service

## Abstract

**Background:**

Large genotyping datasets have become commonplace due to efficient, cheap methods for SNP identification. Typical genotyping datasets may have thousands to millions of data points per accession, across tens to thousands of accessions. There is a need for tools to help rapidly explore such datasets, to assess characteristics such as overall differences between accessions and regional anomalies across the genome.

**Results:**

We present GCViT (Genotype Comparison Visualization Tool), for visualizing and exploring large genotyping datasets. GCViT can be used to identify introgressions, conserved or divergent genomic regions, pedigrees, and other features for more detailed exploration. The program can be used online or as a local instance for whole genome visualization of resequencing or SNP array data. The program performs comparisons of variants among user-selected accessions to identify allele differences and similarities between accessions and a user-selected reference, providing visualizations through histogram, heatmap, or haplotype views. The resulting analyses and images can be exported in various formats.

**Conclusions:**

GCViT provides methods for interactively visualizing SNP data on a whole genome scale, and can produce publication-ready figures. It can be used in online or local installations. GCViT enables users to confirm or identify genomics regions of interest associated with particular traits.

GCViT is freely available at https://github.com/LegumeFederation/gcvit. The 1.0 version described here is available at 10.5281/zenodo.4008713.

## Background

As high throughput genotyping costs have dropped, the dense genotyping of large germplasm collections has become commonplace. Re-sequencing and SNP-array projects are used to identify sequence variants between multiple lines, and may be used to perform genome wide association studies (GWAS) to find variants that are associated with phenotypes. These studies can produce millions of SNPs. For example, Torkamaneh et al. [1] identified 15 million variants among 1007 accessions of soybean, which has relatively low diversity compared with a crop such as maize. Often these data sets are used for a single genome wide association study (GWAS), but such data sets are rich and may be repurposed for other studies. Reuse of this valuable data requires tools for visualization and analysis.

Several tools exist for exploring this data. The command line tool Genotype Query Tools (GQT) [2] and its web form, webGQT [3] provide a means of indexing and querying VCF files. However it lacks visualization options. Many tools are available for genomic and genotypic data visualization [4]. Some of these tools include: Flapjack [5], Integrative Genomics Viewer (IGV) [6], Tassel-GBS [7], JBrowse [8–10], Xena [11], and SNPVersity [12]. These tools provide visualization of selected genomic regions/genes in a single view, but lack a whole genome overview. Tools that provide a whole genome scale visualization in a single view include: CViT (Chromosome Visualization Tool) [13], Synteny Explorer [14], MizBee [15] and SNP & Variation Suite (SVS) Golden Helix® - yet these tools do not automate the comparisons of accessions using SNP data. CViT displays features on “backbones”, including complete genetic and cytogenetic maps, and whole genome views of genomic features. However, although CViT can be integrated into online resources, it is a standalone Perl application that generates static images on predefined comparisons, limiting its utility for interactive exploration.

In this paper we describe a new tool, GCViT (Genotype Comparison Visualization Tool) for dynamic, whole genome visualization of resequencing and SNP array data through histogram, heatmap or haplotype views of two or more accessions selected from a genotyping data set. The visualization enables identification of regions of similarity and difference across the genome. GCViT is built on top of CViTjs, the JavaScript rewrite of CViT. (https://github.com/LegumeFederation/gcvit).

### Implementation

GCViT operates on variant call (VCF) files which have been mapped to a single reference genome assembly. Users select a reference genotype and one or more comparison genotypes, then GCViT performs pairwise comparisons between the comparison and reference genotypes and displays the results on a whole genome view of the reference assembly. GCViT is written in Golang (https://golang.org) and JavaScript. The JavaScript application CViTjs (https://github.com/LegumeFederation/cvitjs) is used to display the comparison results. Resulting images can be downloaded as in PNG or SVG formats.

### Overview

GCViT, CViTjs, and CViT display features across the full genome and use similar glyphs to represent features and similar configuration files. CViT is a generic Perl application for displaying features on any sort of backbone (e.g. linkage group, pseudomolecule) which uses the GD graphics library and produces a static image. CViTjs is a JavaScript rewrite of CViT that makes use of Canvas for drawing images and enables interactive data views. GCViT, a Golang wrapper around CViTjs, provides additional functionality for handling genotype data and permits users to interactively choose reference and comparison genotypes and modify display options.

Although GCViT is able to handle fairly large data sets, data sets of millions of SNPs and/or hundreds of genotypes may need to be subsampled. A utility for accomplishing this, subsample_vcf.pl, is included in the distribution. This script can be used to filter SNPs by quality, and will select representative SNPs within a specified genomic window size.

#### Availability

GCViT is available at https://github.com/LegumeFederation/gcvit under an MIT license. CViTjs is available at https://github.com/LegumeFederation/cvitjs also under an MIT license. The 1.0 version of GCViT that is described here is available at 10.5281/zenodo.4008713.

## Results

### Server-side configuration

An instance of GCViT can be set up in a Docker container or installed as a Go and Nodejs application. Set up consists of the service configuration file, preparation of data sets and connecting them to GCViT, and configuration of the user interface (UI). Instructions for deploying an instance of GCViT are provided in the GitHub repository (https://github.com/LegumeFederation/gcvit).

### The GCViT display

#### Binning and scaling

To handle the display of dense data, the chromosomes are divided up into bins and counts are represented for each bin. The default bin size is 500 kb, but this can be changed in the server-side configuration file and interactively by the user. Bin sizes should be set according to SNP density and the degree of scaling: very high-density genotype data may be suited for smaller bin sizes, but a large genome will require larger bins due to pixel size because data can’t be displayed at a scale less than one pixel.

#### Display types

There are 3 different data displays: histogram, heatmap, and haplotype. The histogram view shows SNP counts in each bin, where the size of each bar represents the count proportional to the minimum and maximum values across the entire genome. The heatmap view shows SNP counts within each bin using color ranges that are proportional to the minimum and maximum values across the genome. The haplotype view shows SNP presence/absence within each bin if the count in the bin matches or exceeds a given threshold.

### User Interface

The user interface (UI) controls are grouped into sections: “Configure View,” where the data set and genotypes are selected; “Display,” where the image and its interactive controls are displayed; and “View Controls,” which contains controls for turning on and off portions of the image. Detailed instructions for the UI are provided in GCViT itself, through the Help button.

#### Selecting the reference genotype

The first step is to select a data set and reference genotype. Data set availability is established in the configuration, along with file paths and data set name. In addition, availability of a particular data set may be controlled via simple authentication. Comparisons can be made only within a single data set.

#### Selecting the comparison genotypes

After selecting the data set and reference genotype, one or more comparison genotypes can be selected and each assigned a distinct color. A full color palette is provided to help distinguish the selected genotypes.

#### SNP comparisons

Comparisons can be displayed on the left or right side of the chromosome backbones. For each comparison, the user selects a display type (histogram, heatmap, or haplotype), and the type of comparison (alleles are different from the reference, same as the reference, or the total SNP count). Depending on the display type, the user has the option of setting specific minimum or maximum values rather than leaving GCViT to calculate them across the genome (histogram and heatmap), or of setting a threshold value (haplotype).

#### Optional settings

In the Configure View Options section, the image can be given a title, the bin size can be changed, the ruler placement can be modified, and the ruler interval (frequency of tic marks and how often coordinate counts are displayed) can be changed.

#### Control buttons

There are three main buttons, Display, Download, and Help. The Display button generates the image. The image may be larger than the viewport, in which case it can be moved by clicking and dragging the image. The Download button gives the option of downloading the results in SVG or PNG formats. There are some differences between the two options: the SVG format is downloaded as the whole image (which may be larger than what is displayed on the screen, while the PNG format will only download what is currently visible in the viewport. The GFF file that was created and used to draw the visualization can also be downloaded. The Help button provides information about GCViT and instructions for using the interface.

#### Pop up box

Clicking on a glyph in the image will pop up a box that identifies the bin number, chromosome coordinates, the value for each accession and the total value for the bin. The pop up box can be customized by modifying the CViTjs pop up template. Examples of potential customizations include link-outs to other resources, such as the Germplasm Repository Information Network (GRIN) accession page, or to a genome browser. In our example on SoyBase there are linkouts to the SoyBase Gbrowse instance, for exploration of genic features in the bin; and to the Legume Information System “Context Viewer,” which enables examination of synteny among similar genomic regions.

#### Key

Above the image, a key is displayed with the currently displayed genotypes and their respective colors. This key will update only after the Display button has been pressed to update the view.

#### Image controls

**Fig. 1:**
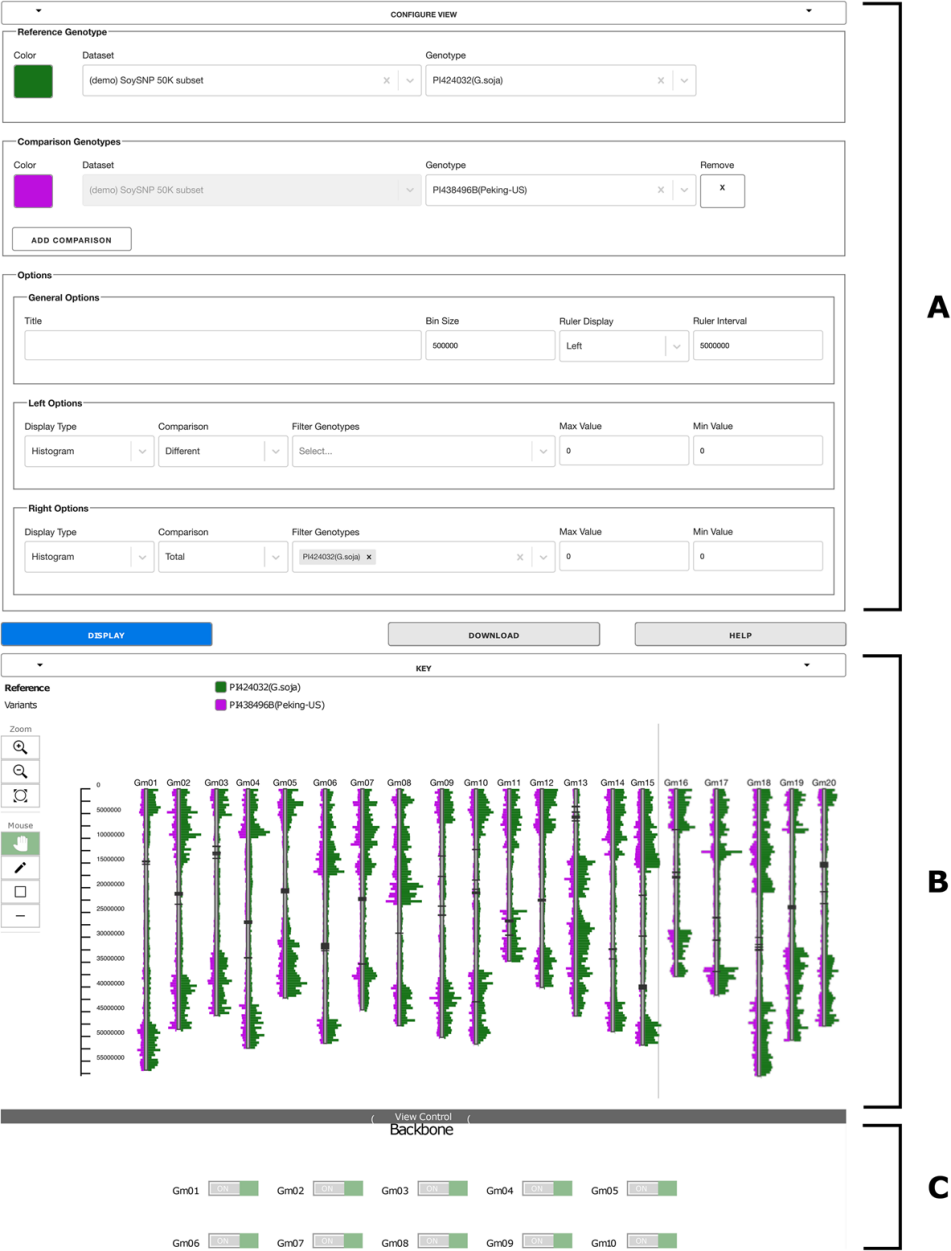
GCViT UI with an example comparison from the SoySNP50K dataset. **a** “configure view” section of the UI in which the figure’s constraints are generated. **b** interactive “display” section of the selected comparison. **c** “view controls” for further filtering of the displayed view

On the left side of the image is a toolbox that provides zoom controls and a set of drawing options that permit drawing free-hand lines or rectangles, an eraser, and a color palette. The image can be moved within the viewport by clicking and dragging with the mouse (Fig. 1). Note that the bin size does not change when zooming in or out.

#### View control

At the bottom of the page, the ‘View Control’ section permits the user to toggle off and on individual chromosomes and other display elements (Fig. 1).

### Data sets

Online instances of GCViT at the time of writing include soybean (https://soybase.org/gcvit/), common bean (https://gcvit.phaseolus.legumeinfo.org), chickpea (https://gcvit.cicer.legumeinfo.org), and peanut (https://peanutbase.org/germplasm/gcvit/). Data sets available for soybean include: the whole U.S. germplasm collection genotyped with the SoySNP50K array [16], resequencing of 481 soybean accessions [17], resequencing of 102 Canadian accessions [18], the soybean Nested Association Mapping (SoyNAM) parents and progeny [19, 20], 222 Korean accessions genotyped using the Axiom® SoyaSNP array [21], 4234 Korean accessions using the Axiom® SoyaSNP array [22], GmHapMap data consisting of 1007 resequenced accessions [1], and genotyping of 374 U.S. and Brazilian accessions [23].

Data available for Chickpea contains genotype information from 279 Chickpea accessions [24]. For common bean, diversity data is available for two diverse collections of *Phaseolus vulgaris*: the Mesoamerican Diversity Panel (MDP) and the Andean Diversity Panel (ADP) [25]. The peanut data set contains the U.S. Peanut Mini Core Collection genotyped using the 58 K Affymetrix SNP array, Axiom Arachis [26].

## Discussion

### Case studies

There are many potential uses for GCViT. Here we describe four use cases.

#### Use case 1: identify introgressions between two common bean populations

**Fig. 2:**
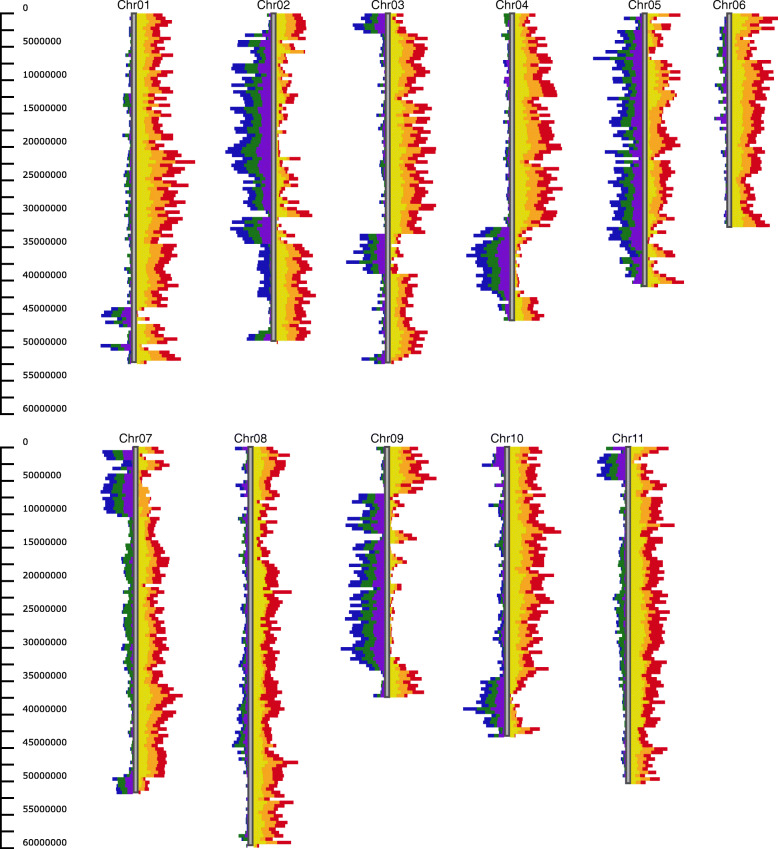
Identify introgression from Mesoamerican population into Andean line Heirloom using the Histogram view with default settings. Differences between Heirloom and Andean lines Dolly (purple), Majesty (green), and Bonus (blue) are displayed on the left-hand side of the chromosome. Differences between Heirloom and Mesoamerican lines Avalanche (yellow), Maverick (orange) and Zorro (red) are displayed on the right-hand side of the chromosome. All of the chromosomes are displayed

Using GCViT on the Bean CAP diversity panels [25], the Andean and Mesoamerican populations can be compared to identify introgressions. In Fig. 2, the Andean line ‘Heirloom’ is the reference genotype, which is compared with three other Andrean lines: Dolly, Majesty and Bonus; and three Mesoamerican lines: Avalanche, Maverick and Zorro. Regions that were introgressed from the Mesoamerican population can be seen on chromosomes 3, 4 and 9. Although there are differences between Heirloom and the other three Andrean lines, there are few differences among the Mesoamerican lines, suggesting that these regions were introgressed from the Mesoamerican population.

#### Use case 2: inheritance analysis

**Fig. 3:**
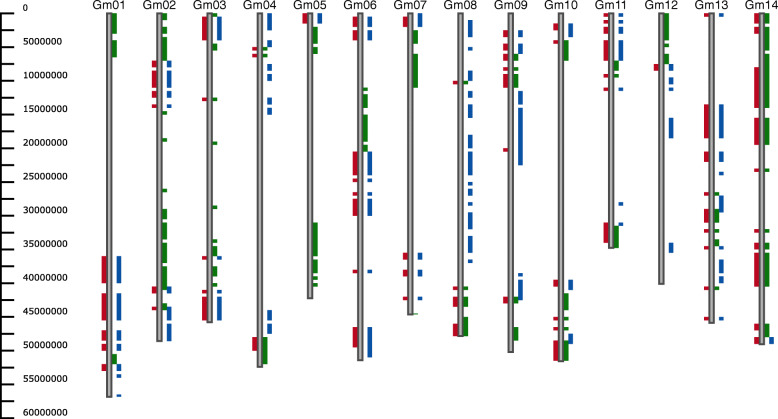
Inheritance of soybean line Blackhawk using the Haplotype view with a threshold of 5. SNP differences between Blackhawk and its sibling Hawkeye (PI 548577) are displayed on the left side of the chromosome in red. SNP differences between Blackhawk and its parents are displayed on the right, with Mukden in green and Richland in blue. The first 14 chromosomes are displayed

Using the SoySNP50K data [16], pedigree relationships were plotted, comparing soybean line Blackhawk (PI 548516) to sibling Hawkeye (PI 548577), and parents Mukden (PI 548391) and Richland (PI 548406) (Fig. 3). In this example, every region with a difference between Blackhawk and its sibling, also shows a difference between Blackhawk and one of its parents, indicating that this region was inherited from different parents in each sibling. From this information, it is apparent that most of Gm04, Gm08, and Gm17 of the siblings were inherited from Mukden, while most of Gm05 was inherited from Richland.

#### Use case 3: identify conserved genomic regions and/or regions of interest

**Fig. 4:**
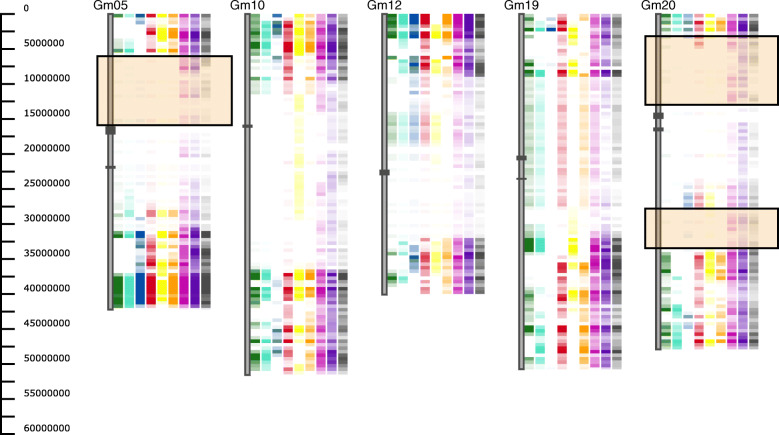
Elite, Landrace and Wild soybean lines were compared against reference accession Williams 82 (Wm82) using the Heatmap view and a Max threshold of 30. In order from left to right are Elite accessions: PI 548655 (green, common name- Forrest), PI 548656 (turquoise, common name- Lee) and PI 546487 (blue, common name-Archer); Landrace accessions: PI 567155A (red), PI 361109 (yellow) and PI 407802 (orange); Wild accessions: PI 407040 (pink), PI 163453 (purple), and PI 407247 (gray). A subset of chromosomes are displayed including Gm05, and Gm20 which show conserved regions within the cultivated soybeans (Elite and Landrace) highlighted in orange boxes. Centromeric regions are indicated by dark gray boxes on the chromosomes

Using the SoySNP50k data, we can identify regions that are conserved between cultivated soybean lines (*Glycine max*) and its wild relative (*Glycine soja*). In this example we used soybean cultivar Williams 82 (Wm82) as the reference and compared it to 6 other cultivated soybean lines and 3 wild (*G.soja*) lines. On chromosome Gm05 and Gm20 there are regions that show no differences between Wm82 and the other cultivated soybeans, but clear differences between the cultivated soybeans and the wild soybeans (Fig. 4). These regions could indicate regions that were selected during domestication.

#### Use case 4: identify if two soybean accessions labeled the same are indeed the same

It is known that there can be genomic variation between soybean cultivars with the same name due to differential segregation of polymorphic regions during the breeding process [27]. One cultivar where we see this variation is the representative soybean genome, Williams 82 (Wm82). In these two examples we show two different situations where two accessions with the same name are not 100% genetically similar.

#### Example 1

**Fig. 5:**
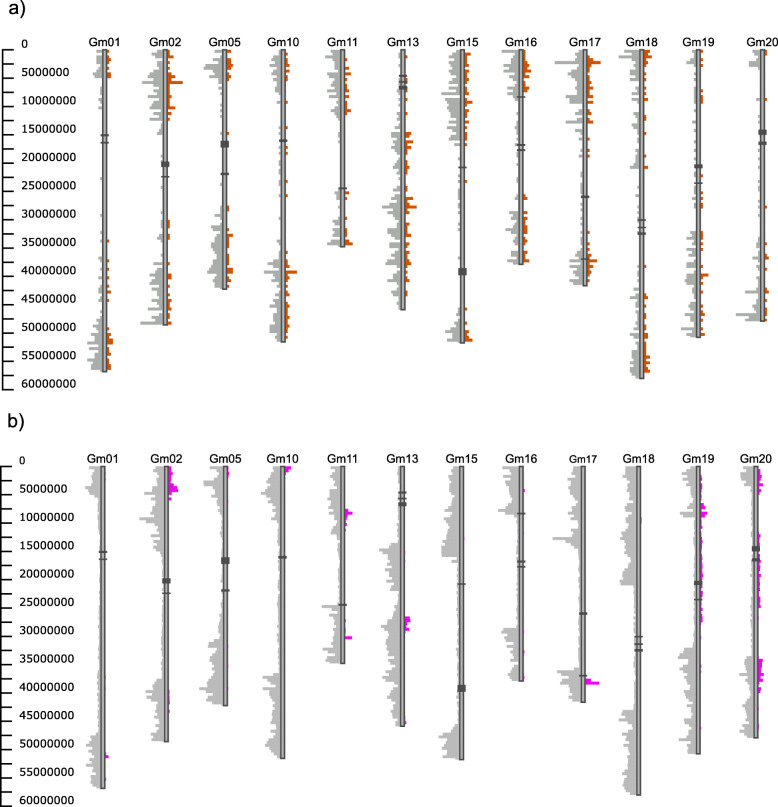
Differences between two lines with the same name using the Histogram view. **a** using a merged dataset of the SoySNP50K and 180 Axiom array from Lee et al. [21] we compared PI 424032 from the SoySNP50K to PI 424032 from Lee et al. SNPs in gray (left) represent the total number of SNPs used in the comparison while SNPs in orange (right) indicate differences between the two lines. **b** using the SoySNP50K dataset, two Dwight lines were compared. Dwight from Dr. Song’s lab and Dwight directly from the soybean germplasm collection. SNPs in gray (left) represent the total number of SNPs used in the comparison while SNPs in pink (right) indicate differences between the two lines. Centromeric regions are indicated by dark gray boxes on the chromosomes. A subset of chromosomes are displayed

In this example we use a soybean accession which was genotyped by two different studies. The two VCF files were merged using BCFtools and a similarity matrix was created using SNPRelate. Accessions overlap was identified and all of the accessions matched their counterpart, except for two. One of these accessions was PI 424032, which was found to have a similarity score of 0.715. The differences between these two accessions were then plotted using GCViT (Fig. 5a). In this example we can see that the line PI 424032 genotyped from the SoySNP50K and Lee et al. [21] are completely different. (Fig. 5a) It was later confirmed by the author/PI (personal comm. Dr. Soon-Chun Jeong) that the wrong seed was received from the Soybean Germplasm Repository.

#### Example 2

Using two different lines of soybean accession Dwight, we are able to identify regions of selection. Using information from the SoySNp50K data set the similarity score was calculated between Dwight and PI 597386. The similarity score between these two lines is 0.977. Using GCViT we can plot where these two lines differ (Fig. 5b) This information shows that these two lines started out the same, but were then grown out for multiple generations in different labs (personal comm. Dr. Qijian Song).

### Resources

A video tutorial can be found here: https://www.youtube.com/watch?v=B2gPVUipWo0 and a blog post on GCViT can be found here: https://www.legumefederation.org/en/blog/2020/03/26/genotype-comparison-visualization-tool-gcvit/

### Further development

GCViT remains in active development. As it is in the process of being adopted for additional organisms and research communities, we are receiving requests for enhancements and new features. These and future requests will be considered for inclusion in subsequent releases.

## Conclusions

GCViT provides useful visualization of SNP data on a whole genome scale. This visualization can provide many insights. Images can be downloaded as publication-ready figures.

## Availability and requirements

**Project name:** GCViT (Genotype Comparison Visualization Tool)

**Project home page:** https://github.com/LegumeFederation/gcvit

**Operating system(s):** Platform Independent

**Programming language:** Golang, JavaScript

**Other requirements**: NodeJS > = 10.13.0 and Go > = 1.10 or Docker to build

**License:** MIT

**Any restrictions to use by non-academics:** No Restrictions

## Data Availability

GCViT is freely available here: https://github.com/LegumeFederation/gcvit. The 1.0 version described here is available at 10.5281/zenodo.4008713. All data used in on-line versions of GCViT can be found at the Legume Federation Datastore.

## Abbreviations

CViT: Chromosome Visualization Tool
GCViT: Genotype Comparison Visualization Tool
GRIN: Germplasm Repository Information Network
GWAS: Genome Wide Association Study
LIS: Legume Information System
SoySNP50k: Illumina Infinium BeadChip containing 50,000 SNPs from soybean, used to sequence the full U.S. soybean germplasm collection.
UI: User Interface
VCF: Variant Call Format
Wm82: Williams 82
